# Carotenoid dynamics in Atlantic salmon

**DOI:** 10.1186/1741-7007-4-10

**Published:** 2006-04-18

**Authors:** Hannah Rajasingh, Leiv Øyehaug, Dag Inge Våge, Stig W Omholt

**Affiliations:** 1 Centre for Integrative Genetics (CIGENE) and Department of Animal and Aquacultural Sciences, Norwegian University of Life Sciences (UMB), 1430 Ås, Norway; 2 Centre for Integrative Genetics (CIGENE) and Department of Chemistry, Biotechnology and Food Sciences, Norwegian University of Life Sciences (UMB), 1430 Ås, Norway

## Abstract

**Background:**

Carotenoids are pigment molecules produced mainly in plants and heavily exploited by a wide range of organisms higher up in the food-chain. The fundamental processes regulating how carotenoids are absorbed and metabolized in vertebrates are still not fully understood. We try to further this understanding here by presenting a dynamic ODE (ordinary differential equation) model to describe and analyse the uptake, deposition, and utilization of a carotenoid at the whole-organism level. The model focuses on the pigment astaxanthin in Atlantic salmon because of the commercial importance of understanding carotenoid dynamics in this species, and because deposition of carotenoids in the flesh is likely to play an important life history role in anadromous salmonids.

**Results:**

The model is capable of mimicking feed experiments analyzing astaxanthin uptake and retention over short and long time periods (hours, days and years) under various conditions. A sensitivity analysis of the model provides information on where to look for possible genetic determinants underlying the observed phenotypic variation in muscle carotenoid retention. Finally, the model framework is used to predict that a specific regulatory system controlling the release of astaxanthin from the muscle is not likely to exist, and that the release of the pigment into the blood is instead caused by the androgen-initiated autolytic degradation of the muscle in the sexually mature salmon.

**Conclusion:**

The results show that a dynamic model describing a complex trait can be instrumental in the early stages of a project trying to uncover underlying determinants. The model provides a heuristic basis for an experimental research programme, as well as defining a scaffold for modelling carotenoid dynamics in mammalian systems.

## Background

Carotenoids are conjugated double-bond pigment molecules synthesized by plants and some bacteria, algae and fungi. Their primary function in these organisms is to absorb light during photosynthesis and to aid in photoprotection [1]. They are classified by structure as carotenes and xanthophylls, depending upon their oxygen and hydrocarbon content. In the animal kingdom, carotenoids are heavily utilized as a source for pigmentation, as vitamin A precursors, and probably also for improving intercellular communication, enhancing immune response, and as antioxidants *in vivo *[2,3]. There is still a limited understanding of the fundamental processes regulating how carotenoids are absorbed and metabolized in mammalian as well as non-mammalian systems. Here we present a dynamic model to describe and analyse the uptake, deposition, utilization and metabolism of a carotenoid at the whole-organism level, Atlantic salmon (*Salmo salar*) being the system chosen.

The carotenoid dynamics in Atlantic salmon is particularly interesting because other marine fish species feeding partly on the same prey in the same marine habitat do not accumulate carotenoids in their muscle, while otherwise having a very similar carotenoid uptake, metabolism and utilization pattern [4,5]. A deeper understanding of how metabolic systems are changed according to changes in selection pressures is of considerable evolutionary interest, since it links mechanistic and life-history reasoning into more comprehensive evolutionary explanations. In addition, a better understanding of carotenoid dynamics is likely to be of substantial economic importance. In commercial salmon production, astaxanthin has proved to be the most efficient carotenoid for muscle pigmentation [6,7], and synthetic astaxanthin is added to feeds in order to make up for the lack of a natural dietary source of the pigment. The retention of dietary carotenoids by the muscle tissues varies from 2 – 22% in salmonid species [6,7], and astaxanthin is responsible for 15–20% of the total feed cost [8].

Even though a model describing the flow and deposition of a carotenoid at the whole-organism level in salmonids has to be specific, it is general enough to function as a scaffold for modelling carotenoid dynamics in mammalian systems. In this way it may contribute to improved understanding of the bioavailability of carotenoids in humans, a trait of considerable importance to human health and disease prevention [9].

### Link between astaxanthin metabolism and fat metabolism

Two basic premises underlying the description to follow are that (i) astaxanthin uptake, transport and delivery are closely associated with fatty acid uptake, transport and delivery, and (ii) major features of astaxanthin absorption, metabolism and transport in salmon are similar to the absorption, metabolism and transport of xanthophylls in mammalian systems. There is some solid evidence for this. Due to their hydrophobic nature, carotenoids are known to be closely associated with fatty acids and transported along with them through the intestine and blood [10,11]. This seems also to be the case for astaxanthin as radioactive studies show that it is associated with all the serum lipoproteins [12], which are the main transporters of esterified fatty acids. Likewise, it is also associated with serum albumin [12], the main transporter of unesterified (or free) fatty acids in mammalian systems [13,14]. Further, it has been found that pigmentation in salmon is quite strongly affected by dietary fat. Higher levels of astaxanthin deposition and retention in rainbow trout [15] and Atlantic salmon [16] have been obtained by increasing dietary lipid levels. This association has been observed in humans as well, where adequate dietary fat levels are necessary for optimal carotenoid absorption [9].

**Figure 1:**
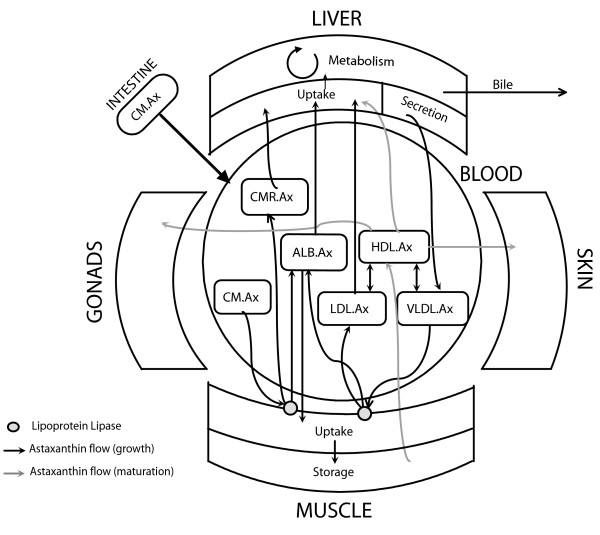
**Astaxanthin uptake, transport and deposition**. The figure illustrates our current understanding of the astaxanthin pathway in salmonids. The uptake of astaxanthin from the intestine, transport in the blood and deposition in the muscle by the various lipoproteins is depicted. During sexual maturation/spawning migration (gray lines), the astaxanthin is redistributed from the muscle to the skin and gonads by the HDL.

Within this framework the presentation to follow is our current understanding (or meta-hypothesis) of the flow and fate of astaxanthin in Atlantic salmon (Figure 1), and our mathematical model (see Methods) attempts to be a representation of this understanding.

### Uptake and transport into blood

Being fat-soluble, dietary astaxanthin is assumed to be in micellar form in the intestine, together with bile salts, fatty acids, monoglycerides and other fat-soluble vitamins [17]. It is believed to passively diffuse into the intestinal lumen, together with fatty acids [11], and the uptake seems to be a slow process taking between 18 and 30 hours [18,19]. The fatty acids are converted into triacyl glycerols (TAG), and the astaxanthin, like other xanthophylls [20], is incorporated together with TAGs in lipoprotein spheres called chylomicrons. These are then transported into the blood [21] and due to its polarity, astaxanthin is assumed to be attached to the surface of the chylomicron spheres [20].

In mammals, transport of chylomicrons from the intestinal lumen into the blood is carried out through lymphatic vessels. While being transported along the blood stream, chylomicrons undergo hydrolysis by lipoprotein lipase (LPL), a triacylglycerol lipase found on the surface of endothelial cells of the tissue capillaries, to yield free fatty acids. The fatty acids and monoglycerols that are derived from chylomicrons in this way are subsequently taken up by the tissues or serum albumin [22,23]. Changes in the lipid composition of a chylomicron modify the affinity of the associated lipoproteins for its surface, causing the chylomicron to change its apolipoprotein signature [24]. When the chylomicrons have lost about 80% of their initial TAG content [22], they become small enough to pass through the endothelium in the liver, and in addition their apolipoprotein signature can then be recognized by specific receptors in the liver [23].

The fate of chylomicron-associated xanthophylls is poorly understood in mammals as well as in fish. It is hypothesized that non-triglyceride components of the chylomicron, including surface molecules such as xanthophylls, may be taken up by extra-hepatic tissues or transferred to other blood lipoproteins [3]. When the chylomicrons reach the liver, however, they still contain a considerable amount of their original carotenoid content [9,11].

Since fish do not seem to have a lymph system similar to the mammalian one, the chylomicrons are assumed to be transported through the primary blood vessels in the intestine [25]. Aside from this, there is reason to assume that chylomicron-based astaxanthin transport and delivery in salmon are quite similar to that in mammals, as discussed above.

Studies in salmon have provided evidence that astaxanthin is strongly associated with a protein likely to be serum albumin [12,26]. We know that serum albumin associates with the surface of lipoprotein particles [22], and we also know that albumin is the major transporter of free fatty acids released during lipolysis by LPL to tissues (including liver and muscle) [13,22]. This suggests that albumin may acquire astaxanthin from chylomicrons during lipolysis as well as directly from chylomicrons in the bloodstream.

### Liver metabolism and excretion

The liver is the main metabolic and excretory organ for carotenoids [27,28] and is considered to have the major responsibility for the metabolic loss of astaxanthin [29]. The liver secretes bile into the intestine to aid in lipid digestion as well as in the excretion of metabolic breakdown products, and radioactive labelling studies with canthaxanthin [27] found bile radioactivity levels to be 8 times higher than the level in blood. The astaxanthin metabolites in the bile are secreted into the intestine and re-absorbed [28]. Radiolabeling experiments also indicate that either astaxanthin or its metabolites are excreted by the kidneys of salmon and rainbow trout [4,27]. The liver is therefore either catabolizing astaxanthin to other pigments or to metabolites that no longer have a chromophore [29].

The exact process of astaxanthin metabolism in the liver is unknown, as is the case with beta-carotene metabolism [11]. We do know, however, that the chylomicron-delivered astaxanthin that is not metabolized is repackaged into very low-density lipoproteins (VLDL) before being sent out into the blood once more [24].

### Transport and deposition in muscle

Despite numerous studies, the mechanism by which free fatty acids enter cells remains poorly understood [30]. We do know that through the action of LPLs in the tissue capillary walls the VLDLs are broken down into low density lipoproteins (LDLs) [23]. Astaxanthin association with LDLs has been observed in salmon [12] and rainbow trout [31], suggesting that astaxanthin-containing LDLs may contribute substantially to the LPL-mediated uptake of astaxanthin by circulating albumin. Astaxanthin is then assumed to be brought to the muscle by circulating albumin [22]. Binding to the muscle cell wall is thought to be non-specific and saturable [32].

After having entered the muscle cell, astaxanthin is deposited in the myotome and binds to actomyosin by weak hydrophobic bonds, forming a complex. The presence of hydroxyl and keto groups at the *β*-end of the carotenoid increases the binding strength [33], explaining the higher deposition of astaxanthin compared to other carotenoids in salmon. Metabolites of astaxanthin have also been found in the connective tissues between myotomes [28].

During VLDL and LDL flow through the blood, some of the TAG found in these lipoproteins is transferred to high-density lipoprotein packets (HDL) by the cholesteryl ester transfer protein (CETP) [22,34]. Given that astaxanthin is transported along with the TAGs to the HDLs, this mechanism is likely to explain the observed high levels of HDL-associated astaxanthin in immature salmon [12], where there is no pigment transport out of the muscle.

An interesting feature here is that unlike in mammalian systems, Atlantic salmon muscle has been found to express albumin [35]. Whether this has any significance on astaxanthin retention/transport is yet to be determined.

### Release from muscle

There is no detectable reduction in astaxanthin levels in the flesh even after several weeks of starvation in the sexually immature salmon [36,37]. Sexually maturing salmonids, however, transfer flesh carotenoids to the skin and gonads, and there is a prominent loss of whole-body carotenoids in this stage [38]. HDL seems to be the major transporter of astaxanthin from muscle to the skin [12]. In female fish, astaxanthin is also transported from muscle and the gastro-intestinal tract to the ovaries by the lipoprotein vitellogenin, which is produced by the liver [39]. In the skin, the highest levels are found in the dorsal cutis, indicating an association with the melanin that is found there [28]. HDL has also been reported to transport astaxanthin from the skin to the gonads and eggs [19]. In the eggs it is bound to lipovitellin, the predominant egg yolk lipoprotein derived from vitellogenin [40]. The eggs seem to require a minimum level of astaxanthin in order to be viable [41], though this is still under debate [42].

## Results

Our current conception of carotenoid metabolism as illustrated in the previous section was translated into a differential equations model (see Methods). Model predictions were then tested against available experimental data.

### Single pulse feeding

**Figure 2:**
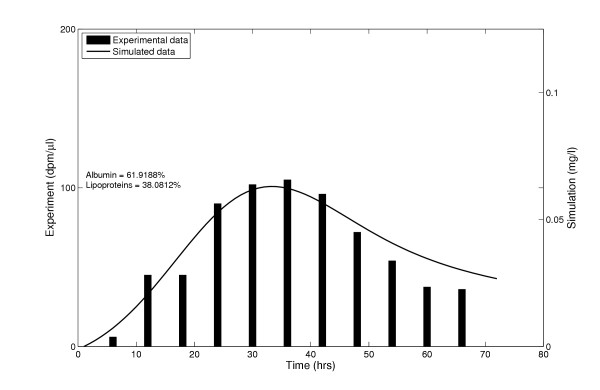
**Astaxanthin plasma levels after a single feeding**. The experimental data of Aas *et al. *plotted with the corresponding model-simulated results. The change in plasma level of astaxanthin over 72 hrs is shown, after a single feeding.

After some preliminary calibration (see Methods) we first tested whether the model was capable of mimicking the temporal blood level pattern of astaxanthin observed in a single pulse feeding experiment conducted by Aas *et al. *[12]. In this experiment Atlantic salmon weighing 700 g were force fed a single dose of ^14^C-astaxanthin and the radioactivity level in the blood was measured over a 72 hour period. The total blood level peaked around 30 hrs, with contributions from the various lipoprotein fractions as well as a high-density protein fraction (~57.7%) assumed to be albumin. In the simulation the weight of the fish and hence the tissue weights were kept constant. Although the parameter freedom in this first simulation is considerable, it is still encouraging that the model proved able to mimic the experimental data quite well at such a high time resolution (Figure 2).

### Long-term interval feeding

**Figure 3:**
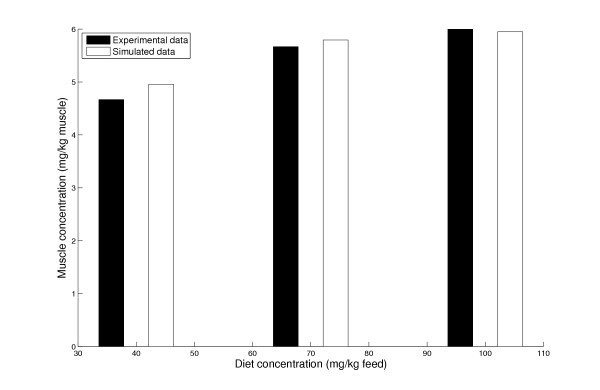
**Muscle uptake at different dietary concentrations of astaxanthin**. The experimental data of March *et al. *plotted with the corresponding model-simulated results. The muscle astaxanthin concentrations after a period of 40 weeks of feeding astaxanthin at dietary levels of 40, 70 and 100 mg/kg feed are shown.

The model was then expanded to predict astaxanthin accumulation in the flesh over a much longer time span (> 1 year), with constant interval feeding and accounting for the associated weight increase of tissues. Except for time scale adjustments, the same set of parameter values was used as in the previous case (see Table 1). We chose a feed study done by March and Macmillan [43] as our experimental test bed. In this experiment development of the carotenoid level in the flesh of Atlantic salmon was studied as a function of different dietary levels of astaxanthin (40, 70 and 100 mg/kg feed) over a period of 40 weeks. It was observed that when fish of initial weight 200 g were fed for 40 weeks, the pigment level in the muscle showed clear signs of saturation as a function of the astaxanthin level in the food. Even though we did not change any of the parameter values made use of in the single pulse feeding experiment, the model was able to mimic the temporal astaxanthin concentration in the flesh as well as the saturation effect quite neatly (Figure 3).

### Quasi-steady state sensitivity analysis

A sensitivity analysis of the model was performed in order to identify which processes described in the model were the least robust to changes in parameter values. The actual system is non-autonomous due to the time-dependent weight *w*, but due to the slow increase of *w *compared to the time-scales of feeding and metabolism, we assumed in the analysis that the system is close to its quasi steady state (QSS) at any time. The rationale for this analysis is that it discloses which parameter values are really influencing the quantitative predictions of the model. If these parameter values are not experimentally well confined, one needs to estimate them more accurately by experimental means before one can claim that the model provides a good representation of the system under study.

**Figure 4:**
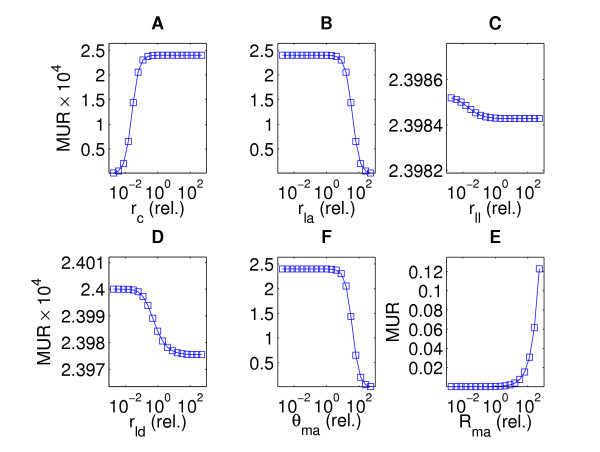
**Sensitivity analysis of the model to variation in parameters – QSS**. Plots of the sensitivity of the muscle uptake rate MUR to variation in the log of key parameters of the model equations; sensitivity to variation in rate of uptake into chylomicrons from intestine, *r*_*c *_(A), rate of deposition in the liver by albumin, *r*_*la *_(B), rate of deposition in the liver by LDL, *r*_*ll *_(C), rate of metabolic degradation in the liver, *r*_*ld *_(D), rate of uptake into the muscle from albumin, *R*_*ma *_(E) and the threshold of saturation in the muscle, *θ*_*ma *_(F). The values of these parameters are relative to the values given in Table 1.

The analysis shows that the uptake in the muscle (MUR) is most sensitive to changes in the rate of deposition of albumin-bound astaxanthin (*r*_*ma*_), as well as to the threshold of saturation (*θ*_*ma*_) (Figure 4). The uptake and degradation in the liver (*r*_*la*_, *r*_*ll*_, *r*_*ld*_) does not seem to affect muscle deposition to any significant extent. Though the muscular uptake seems to be proportional to the intestinal uptake (*r*_*c*_), this is the case only until it saturates, at which point increasing the uptake from the intestine would not have any effect on the uptake in the muscle.

Assuming that the model provides a good representation of the underlying biology, such an analysis is also a means of finding possible sites where the system is sensitive to genetic variation. In this case, the results suggest that genetic variation associated with processes influencing the uptake rate of astaxanthin over the muscle membrane seems to be of particular importance.

### Time-series sensitivity analysis

The parameters of the model were analysed for sensitivity using the data from the single pulse feeding experiment [12] to determine which of the parameters affect the dynamics of the model output. Given the large freedom in possible parameter values, this analysis mainly focused on finding out whether the parameters had to lie within a certain range in order to obtain a good fit with the experimental data.

**Figure 5:**
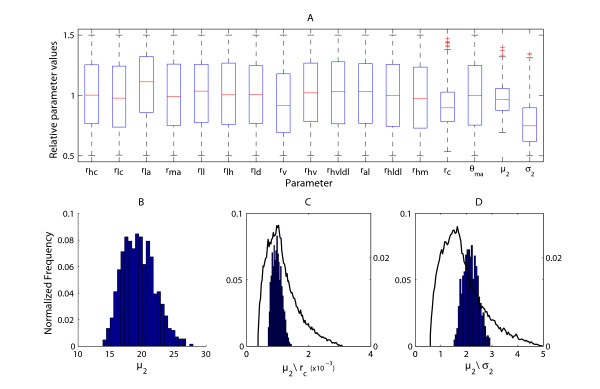
**Sensitivity analysis of the model to variation in parameters – Time-series**. (A) – Boxplot of the selected 1,264 parameter sets out of the total 50,000, which have values giving a total sum of squared deviation of 30% or less from the experimental data, as well as a relative deviation of 50% or less at each individual experimental time point. (B) – Distribution of parameter *μ*_2 _for the 1264 selected parameter sets. (C) – Distribution of the ratio *μ*_2_/*r*_*c *_for the 1,264 selected parameter sets (bars, left y-axis) as well as the distribution of the ratio for all 50,000 parameter sets (curve, right y-axis). (D) – Distribution of the ratio *μ*_2_/*σ*_2 _for the 1,264 selected parameter sets (bars, left y-axis) as well as the distribution of the ratio for all 50,000 parameter sets (curve, right y-axis).

The analysis shows that the most influential parameters, *i.e. *those having the smallest range of values which give a good fit, are those determining the time delay occurring during intestinal uptake of astaxanthin (mean *μ*_2 _standard deviation *σ*_2 _of the integral kernel, see Methods) and the bioavailable fraction of astaxanthin (*r*_*c*_)(Figure 5A). This is to be expected as the time and rate at which the astaxanthin enters the blood stream and hence the time at which the blood astaxanthin concentration peaks are dependent on these three parameters. The rest of the parameters have a more uniform range, indicating that the model is quite robust to small changes in their values.

The interaction between the three selected parameters was then analysed further by determining the distribution of their ratios. There is a clear narrowing in the range of possible values (Figures 5B, 5C, 5D) which will produce a curve that fits the data well.

### Regulatory control of astaxanthin release from the muscle?

During the spawning migration the astaxanthin stored in the flesh is redistributed to the skin and ovaries in female fish and to the skin in males [38,44]. There is no doubt that the astaxanthin release from the muscle is directly or indirectly under hormonal influence as sexual immature salmon do not release astaxanthin to the blood even when they are starved for several weeks [36,37]. It has to be decided, however, whether this release is under specific regulatory control or not, *i.e. *does the salmon possess a specific regulatory apparatus responsible for the release of astaxanthin from its actomyosin binding sites and its subsequent transport to and through the muscle membrane to the blood?

**Figure 6:**
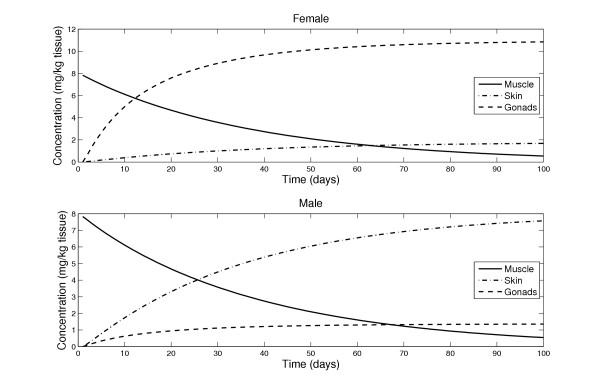
**Astaxanthin redistribution induced by sexual maturation**. Model prediction of the redistribution of astaxanthin from the muscle to the skin and gonads in male and female fish during sexual maturation and spawning migration. Simulations were performed for a period of 100 days of starvation, *i.e. *no input feeding and tissue weight loss.

Based on available data on the muscle degradation rate of salmon during migration and spawning [45], we developed a modified version of the model presuming that the release of astaxanthin from the muscle to the blood is proportional to the muscle degradation rate, as obtained from literature [46]. No input astaxanthin was given in the simulations and the initial muscle concentration was set to that obtained after simulating a year of feeding. We found that the release rate of astaxanthin due to muscle degradation can account for observed release of astaxanthin in sexually mature salmon (Figure 6). We therefore predict that a specific regulatory system controlling the release of astaxanthin from the muscle does not exist. The release of the pigment into the blood is according to this hypothesis a side effect of androgen-initiated autolytic degradation of the muscle [47]. Having entered the blood stream, astaxanthin becomes associated with HDL particles [48] in the blood, where the skin and gonads take some of it up, and the rest is metabolized by the liver [38]. The above explanation suggests that muscle functions as an astaxanthin 'sink' in the sea and as a 'source' in the river.

## Discussion

The model draws upon the general biochemistry of carotenoids, fish physiology and fatty acid metabolism in vertebrates and is capable of mimicking key experiments. One may object that this capability is not due to a valid representation of the system, but is caused by the lack of experimental data giving too much freedom in the choice of parameter values. However, what we have provided here is not a high-resolution prediction machine, but a typical representative of an early-phase model where the experimental data allows the construction of a tentative model, but does not permit extensive confirmation of underlying premises and parameter values. The heuristic value of such models should not be underestimated. The development as well as the subsequent analysis of models of this type are very instrumental for identifying which assumptions need closer experimental attention and which kind of data that should be collected in order to get a more solid and comprehensive understanding of the system. For example, from the sensitivity analysis it can be seen that the muscle uptake process needs to be understood and quantified in a more explicit manner.

Elucidating the regulatory pathways and processes underlying a complex polygenic trait and finding the genetic polymorphisms that are responsible for observed phenotypic variation are two major challenges currently confronting production biology and biomedicine. We have shown that a mathematical model may contribute substantially to both aspects by integrating available empirical data into a concerted whole, by identifying which processes are most sensitive to variation in parameter values and by identifying those that are not likely to be under strict regulatory control.

## Conclusion

We have translated the available knowledge of carotenoid metabolism in vertebrates into a specific dynamic model of astaxanthin uptake, transport, utilization and metabolism in Atlantic salmon. The model provides a theoretical framework for a quantitative understanding of the factors underlying observed phenotypic variation in flesh carotenoid concentration. It identifies the uptake process over the muscle membrane as a potentially important source of variation. It predicts that there is no specific regulatory system for the release of astaxanthin from the muscle cells into the blood stream. Finally, the model provides a scaffold for modelling carotenoid metabolism in other vertebrate systems for epistemic as well as instrumental purposes.

## Methods

### The model

The uptake, transport and deposition of astaxanthin in the salmon was modelled as a function of the feed input and the feeding frequency from when the fish is around 100 grams, until it is slaughtered at day *N*. The dimension of the time unit *t *is denoted as *tu*, which can be either hours or days depending on the simulations.

The intake of astaxanthin is a function of the amount of astaxanthin in the feed *A*_*f *_(dimension-*mg*·(*kg feed*)^-1^) and the feed intake *F*_*i *_(*kg feed*·*tu*^-1^). In a domesticated situation, the feed intake is in turn a function of the weight of the fish *FW *(*kg*), the feeding frequency *F*_*f *_(*tu*^-1^) and the Feed Conversion Ratio *FCR*. The rate of intake of astaxanthin *I*_*ax*_(*t*) (*mg*·*tu*^-1^) can then be described by the function

*I*_*ax*_(*t*) = *A*_*f *_*F*_*i*_(*FW*_*T*_, *F*_*f*_, *FCR*).

The weight of the fish *FW*_*T *_at the beginning of a given day *T*, *T *∈ {0, 1,...*N*}, is stipulated by use of specific growth rate *SGR *data;

FWT=FWT−1(1+SGRT100),
 MathType@MTEF@5@5@+=feaafiart1ev1aaatCvAUfKttLearuWrP9MDH5MBPbIqV92AaeXatLxBI9gBaebbnrfifHhDYfgasaacH8akY=wiFfYdH8Gipec8Eeeu0xXdbba9frFj0=OqFfea0dXdd9vqai=hGuQ8kuc9pgc9s8qqaq=dirpe0xb9q8qiLsFr0=vr0=vr0dc8meaabaqaciaacaGaaeqabaqabeGadaaakeaacqWGgbGrcqWGxbWvdaWgaaWcbaGaemivaqfabeaakiabg2da9iabdAeagjabdEfaxnaaBaaaleaacqWGubavcqGHsislcqaIXaqmaeqaaOWaaeWaaeaacqaIXaqmcqGHRaWkdaWcaaqaaiabdofatjabdEeahjabdkfasnaaBaaaleaacqWGubavaeqaaaGcbaGaeGymaeJaeGimaaJaeGimaadaaaGaayjkaiaawMcaaiabcYcaSaaa@42E6@

where *SGR*_*T *_the specific growth rate at day *T *and is empirically obtained [49]. To get a correspondence between the algebraic weight equation and the continuous differential equations model presented below, weight at time *t *(*W*(*t*)) is defined as the weight at the end of the previous day, i.e. *W*(*t*) = *FW*_*T*_.

In order to express state variables and parameters as per ml blood, per kg liver or per kg muscle, we assumed the blood volume (*B*_*v*_(*t*)), the liver weight (*L*_*w*_(*t*)), and the muscle weight (*M*_*w*_(*t*)) to make up approximately 4.5%, 1% and 50% of the total fish weight, respectively [50,51]. (The tissue weights are related to the overall body weight by the allometric relationships: *B*_*v*_(*t*) = *bW*(*t*)^*γ*^, *L*_*w*_(*t*) = *lW*(*t*)^*λ *^and *M*_*w*_(*t*) = *mW*(*t*)^*ρ*^, with *γ*, *λ *and *ρ *usually not being equal to 1, but the error being considered negligible here, we set them as 1).

#### a. Ordinary differential equations

Mathematical modelling by ordinary differential equations (ODEs) is a standard means to describe and analyse dynamic biological processes in continuous time, e.g. [52-55]. Here, the flow and uptake of astaxanthin through the system is represented using eight ordinary differential equations. The linear uptake and transport terms in the equations contain rates (denoted by small *r*) that have a dimension of *tu*^-1^.

The relative rate of change of the concentration of astaxanthin bound to chylomicrons in the blood stream (*X*_*cm*_(*t*)), is dependent upon the flow of astaxanthin through the intestinal wall, the rate of uptake of astaxanthin from the chylomicron surface by serum albumin, and the rate of LPL-mediated release of astaxanthin (which is taken up by muscle as well as causing creation of chylomicron remnants) and is given by

X˙cm︸(mg⋅(ml blood)−1⋅tu−1)=rcIaxi(t)Bv(t)−1︸Rate of uptake ofchylomicron-bound Axinto blood−ϕrhcXcm︸Rate of uptake of chylomicron-bound Axinto albumin due to LPL−(1−ϕ)rhcXcm︸Rate of conversion of chylomicron-bound Ax intochylomicron remnants.
 MathType@MTEF@5@5@+=feaafiart1ev1aaatCvAUfKttLearuWrP9MDH5MBPbIqV92AaeXatLxBI9gBaebbnrfifHhDYfgasaacH8akY=wiFfYdH8Gipec8Eeeu0xXdbba9frFj0=OqFfea0dXdd9vqai=hGuQ8kuc9pgc9s8qqaq=dirpe0xb9q8qiLsFr0=vr0=vr0dc8meaabaqaciaacaGaaeqabaqabeGadaaakeaadaagaaqaaiqbdIfayzaacaWaaSbaaSqaaiabdogaJjabd2gaTbqabaaabaWaaeWaaeaacqWGTbqBcqWGNbWzcqGHflY1daqadaqaaiabd2gaTjabdYgaSjaaykW7cqWGIbGycqWGSbaBcqWGVbWBcqWGVbWBcqWGKbazaiaawIcacaGLPaaadaahaaadbeqaaiabgkHiTiabigdaXaaaliabgwSixlabdsha0jabdwha1naaCaaameqabaGaeyOeI0IaeGymaedaaaWccaGLOaGaayzkaaaakiaawIJ=aiabg2da9maayaaabaGaemOCai3aaSbaaSqaaiabdogaJbqabaGccqWGjbqsdaWgaaWcbaGaemyyaeMaemiEaGNaemyAaKgabeaakmaabmaabaGaemiDaqhacaGLOaGaayzkaaGaemOqai0aaSbaaSqaaiabdAha2bqabaGcdaqadaqaaiabdsha0bGaayjkaiaawMcaamaaCaaaleqabaGaeyOeI0IaeGymaedaaaabaiqabaGaeeOuaiLaeeyyaeMaeeiDaqNaeeyzauMaeeiiaaIaee4Ba8MaeeOzayMaeeiiaaIaeeyDauNaeeiCaaNaeeiDaqNaeeyyaeMaee4AaSMaeeyzauMaeeiiaaIaee4Ba8MaeeOzaygabaGaee4yamMaeeiAaGMaeeyEaKNaeeiBaWMaee4Ba8MaeeyBa0MaeeyAaKMaee4yamMaeeOCaiNaee4Ba8MaeeOBa4Maeeyla0IaeeOyaiMaee4Ba8MaeeyDauNaeeOBa4MaeeizaqMaeeiiaaIaeeyqaeKaeeiEaGhabaGaeeyAaKMaeeOBa4MaeeiDaqNaee4Ba8MaeeiiaaIaeeOyaiMaeeiBaWMaee4Ba8Maee4Ba8MaeeizaqgaaOGaayjo+dGaeyOeI0YaaGbaaeaaiiGacqWFvpGAcqWGYbGCdaWgaaWcbaGaemiAaGMaem4yamgabeaakiabdIfaynaaBaaaleaacqWGJbWycqWGTbqBaeqaaaabaiqabaGaeeOuaiLaeeyyaeMaeeiDaqNaeeyzauMaeeiiaaIaee4Ba8MaeeOzayMaeeiiaaIaeeyDauNaeeiCaaNaeeiDaqNaeeyyaeMaee4AaSMaeeyzauMaeeiiaaIaee4Ba8MaeeOzayMaeeiiaacabaGaee4yamMaeeiAaGMaeeyEaKNaeeiBaWMaee4Ba8MaeeyBa0MaeeyAaKMaee4yamMaeeOCaiNaee4Ba8MaeeOBa4Maeeyla0IaeeOyaiMaee4Ba8MaeeyDauNaeeOBa4MaeeizaqMaeeiiaaIaeeyqaeKaeeiEaGhabaGaeeyAaKMaeeOBa4MaeeiDaqNaee4Ba8MaeeiiaaIaeeyyaeMaeeiBaWMaeeOyaiMaeeyDauNaeeyBa0MaeeyAaKMaeeOBa4MaeeiiaaIaeeizaqMaeeyDauNaeeyzauMaeeiiaaIaeeiDaqNaee4Ba8MaeeiiaaIaeeitaWKaeeiuaaLaeeitaWeaaOGaayjo+dGaeyOeI0YaaGbaaeaadaqadaqaaiabigdaXiabgkHiTiab=v9aQbGaayjkaiaawMcaaiabdkhaYnaaBaaaleaacqWGObaAcqWGJbWyaeqaaOGaemiwaG1aaSbaaSqaaiabdogaJjabd2gaTbqabaaaeaGabeaacqqGsbGucqqGHbqycqqG0baDcqqGLbqzcqqGGaaicqqGVbWBcqqGMbGzcqqGGaaicqqGJbWycqqGVbWBcqqGUbGBcqqG2bGDcqqGLbqzcqqGYbGCcqqGZbWCcqqGPbqAcqqGVbWBcqqGUbGBcqqGGaaicqqGVbWBcqqGMbGzcqqGGaaiaeaacqqGJbWycqqGObaAcqqG5bqEcqqGSbaBcqqGVbWBcqqGTbqBcqqGPbqAcqqGJbWycqqGYbGCcqqGVbWBcqqGUbGBcqqGTaqlcqqGIbGycqqGVbWBcqqG1bqDcqqGUbGBcqqGKbazcqqGGaaicqqGbbqqcqqG4baEcqqGGaaicqqGPbqAcqqGUbGBcqqG0baDcqqGVbWBaeaacqqGJbWycqqGObaAcqqG5bqEcqqGSbaBcqqGVbWBcqqGTbqBcqqGPbqAcqqGJbWycqqGYbGCcqqGVbWBcqqGUbGBcqqGGaaicqqGYbGCcqqGLbqzcqqGTbqBcqqGUbGBcqqGHbqycqqGUbGBcqqG0baDcqqGZbWCaaGccaGL44pacqGGUaGlaaa@63C2@

Here, *r*_*c *_is the bioavailable fraction of provided astaxanthin. Due to the time delay between feeding and the astaxanthin actually entering the intestine, *I*_*axi*_(*t*), the pigment entering the intestinal lumen at time *t*, is expressed as a convolution of the astaxanthin in the feed and the function *Z *(integral kernel), *i.e. *Iaxi(t)=∫−∞∞Iax(t)Z(t−s)ds
MathType@MTEF@5@5@+=feaafiart1ev1aaatCvAUfKttLearuWrP9MDH5MBPbIqV92AaeXatLxBI9gBaebbnrfifHhDYfgasaacH8akY=wiFfYdH8Gipec8Eeeu0xXdbba9frFj0=OqFfea0dXdd9vqai=hGuQ8kuc9pgc9s8qqaq=dirpe0xb9q8qiLsFr0=vr0=vr0dc8meaabaqaciaacaGaaeqabaqabeGadaaakeaacqWGjbqsdaWgaaWcbaGaemyyaeMaemiEaGNaemyAaKgabeaakmaabmaabaGaemiDaqhacaGLOaGaayzkaaGaeyypa0Zaa8qCaeaacqWGjbqsdaWgaaWcbaGaemyyaeMaemiEaGhabeaakmaabmaabaGaemiDaqhacaGLOaGaayzkaaGaemOwaO1aaeWaaeaacqWG0baDcqGHsislcqWGZbWCaiaawIcacaGLPaaacqWGKbazcqWGZbWCaSqaaiabgkHiTGGaciab=5HiLcqaaiab=5HiLcqdcqGHRiI8aaaa@4CB4@. The convolution is used in order to represent the distributed time delay of astaxanthin between when it enters the intestine to when it finally diffuses into the intestinal lumen. For the sake of convenience, we assume *I*_*axi *_and *Z *to be Gaussian functions with means *μ*_1_, *μ*_2 _and standard deviations *σ*_1_, *σ*_2_, respectively. While *I*_*axi *_is assumed to have a small deviation, the mean of the integral kernel *Z *determines the length of the time delay and the deviation determines the spread. The convolution function *I*_*axi *_is then another Gaussian of the form

Iaxi(t)=12π(σ12+σ22)e[t−(μ1+μ2)]2/[2(σ12+σ22)].
 MathType@MTEF@5@5@+=feaafiart1ev1aaatCvAUfKttLearuWrP9MDH5MBPbIqV92AaeXatLxBI9gBaebbnrfifHhDYfgasaacH8akY=wiFfYdH8Gipec8Eeeu0xXdbba9frFj0=OqFfea0dXdd9vqai=hGuQ8kuc9pgc9s8qqaq=dirpe0xb9q8qiLsFr0=vr0=vr0dc8meaabaqaciaacaGaaeqabaqabeGadaaakeaacqWGjbqsdaWgaaWcbaGaemyyaeMaemiEaGNaemyAaKgabeaakmaabmaabaGaemiDaqhacaGLOaGaayzkaaGaeyypa0ZaaSaaaeaacqaIXaqmaeaadaGcaaqaaiabikdaYGGaciab=b8aWnaabmaabaGae83Wdm3aa0baaSqaaiabigdaXaqaaiabikdaYaaakiabgUcaRiab=n8aZnaaDaaaleaacqaIYaGmaeaacqaIYaGmaaaakiaawIcacaGLPaaaaSqabaaaaOGaemyzau2aaWbaaSqabeaadaWcgaqaamaadmaabaGaemiDaqNaeyOeI0YaaeWaaeaacqWF8oqBdaWgaaadbaGaeGymaedabeaaliabgUcaRiab=X7aTnaaBaaameaacqaIYaGmaeqaaaWccaGLOaGaayzkaaaacaGLBbGaayzxaaWaaWbaaWqabeaacqaIYaGmaaaaleaadaWadaqaaiabikdaYmaabmaabaGae83Wdm3aa0baaWqaaiabigdaXaqaaiabikdaYaaaliabgUcaRiab=n8aZnaaDaaameaacqaIYaGmaeaacqaIYaGmaaaaliaawIcacaGLPaaaaiaawUfacaGLDbaaaaaaaOGaeiOla4caaa@6133@

The parameter *r*_*hc *_expresses the rate at which a fraction *φ*, (0 ≤ *φ *= 1) of chylomicron-bound astaxanthin is taken up by serum albumin as well as the rate at which the remaining fraction is converted to chylomicron remnants-bound astaxanthin. Both occur due to the activity of LPL.

The relative rate of change of the concentration of astaxanthin bound to chylomicron remnants (*X*_*cmr*_(*t*)) is then given by the fraction (1-*φ*) of the rate loss term that ends up in chylomicron remnants, minus the liver uptake rate of the remnants;

X˙cmr︸(mg⋅(ml blood)−1⋅tu−1)=(1−ϕ)rhcXcm−rlcXcmr︸Rate of uptakeof chylomicron remnant-boundAx by liver.
 MathType@MTEF@5@5@+=feaafiart1ev1aaatCvAUfKttLearuWrP9MDH5MBPbIqV92AaeXatLxBI9gBaebbnrfifHhDYfgasaacH8akY=wiFfYdH8Gipec8Eeeu0xXdbba9frFj0=OqFfea0dXdd9vqai=hGuQ8kuc9pgc9s8qqaq=dirpe0xb9q8qiLsFr0=vr0=vr0dc8meaabaqaciaacaGaaeqabaqabeGadaaakeaadaagaaqaaiqbdIfayzaacaWaaSbaaSqaaiabdogaJjabd2gaTjabdkhaYbqabaaabaWaaeWaaeaacqWGTbqBcqWGNbWzcqGHflY1daqadaqaaiabd2gaTjabdYgaSjabbccaGiabdkgaIjabdYgaSjabd+gaVjabd+gaVjabdsgaKbGaayjkaiaawMcaamaaCaaameqabaGaeyOeI0IaeGymaedaaSGaeyyXICTaemiDaqNaemyDau3aaWbaaWqabeaacqGHsislcqaIXaqmaaaaliaawIcacaGLPaaaaOGaayjo+dGaeyypa0ZaaeWaaeaacqaIXaqmcqGHsisliiGacqWFvpGAaiaawIcacaGLPaaacqWGYbGCdaWgaaWcbaGaemiAaGMaem4yamgabeaakiabdIfaynaaBaaaleaacqWGJbWycqWGTbqBaeqaaOGaeyOeI0YaaGbaaeaacqWGYbGCdaWgaaWcbaGaemiBaWMaem4yamgabeaakiabdIfaynaaBaaaleaacqWGJbWycqWGTbqBcqWGYbGCaeqaaaabaiqabaGaeeOuaiLaeeyyaeMaeeiDaqNaeeyzauMaeeiiaaIaee4Ba8MaeeOzayMaeeiiaaIaeeyDauNaeeiCaaNaeeiDaqNaeeyyaeMaee4AaSMaeeyzaugabaGaee4Ba8MaeeOzayMaeeiiaaIaee4yamMaeeiAaGMaeeyEaKNaeeiBaWMaee4Ba8MaeeyBa0MaeeyAaKMaee4yamMaeeOCaiNaee4Ba8MaeeOBa4MaeeiiaacabaGaeeOCaiNaeeyzauMaeeyBa0MaeeOBa4MaeeyyaeMaeeOBa4MaeeiDaqNaeeyla0IaeeOyaiMaee4Ba8MaeeyDauNaeeOBa4MaeeizaqgabaGaeeyqaeKaeeiEaGNaeeiiaaIaeeOyaiMaeeyEaKNaeeiiaaIaeeiBaWMaeeyAaKMaeeODayNaeeyzauMaeeOCaihaaOGaayjo+dGaeiOla4caaa@B069@

Here *r*_*lc *_is the relative rate of uptake in liver.

The relative rate of change in the amount of astaxanthin in the blood bound to serum albumin (*X*_*a*_(*t*)) is dependent on the uptake of astaxanthin from chylomicrons, VLDL and LDL, minus the rate of albumin-mediated deposition of astaxanthin in liver and muscle tissues;

X˙a︸(mg⋅(ml blood)−1⋅tu−1)=ϕrhcXcm+ηrhvXvldl︸Rate of uptakeof VLDL-bound Axinto albumin by LPL+ralXldl︸Rate of uptakeof LDL-bound Axby albumin at muscle−rlaXa︸Rate of depositionof Ax into liver−RmaSma(Xa)︸Rate of depositionof Ax into muscle.
 MathType@MTEF@5@5@+=feaafiart1ev1aaatCvAUfKttLearuWrP9MDH5MBPbIqV92AaeXatLxBI9gBaebbnrfifHhDYfgasaacH8akY=wiFfYdH8Gipec8Eeeu0xXdbba9frFj0=OqFfea0dXdd9vqai=hGuQ8kuc9pgc9s8qqaq=dirpe0xb9q8qiLsFr0=vr0=vr0dc8meaabaqaciaacaGaaeqabaqabeGadaaakeaadaagaaqaaiqbdIfayzaacaWaaSbaaSqaaiabdggaHbqabaaabaWaaeWaaeaacqWGTbqBcqWGNbWzcqGHflY1daqadaqaaiabd2gaTjabdYgaSjabbccaGiabdkgaIjabdYgaSjabd+gaVjabd+gaVjabdsgaKbGaayjkaiaawMcaamaaCaaameqabaGaeyOeI0IaeGymaedaaSGaeyyXICTaemiDaqNaemyDau3aaWbaaWqabeaacqGHsislcqaIXaqmaaaaliaawIcacaGLPaaaaOGaayjo+dGaeyypa0dcciGae8x1dOMaemOCai3aaSbaaSqaaiabdIgaOjabdogaJbqabaGccqWGybawdaWgaaWcbaGaem4yamMaemyBa0gabeaakiabgUcaRmaayaaabaGae83TdGMaemOCai3aaSbaaSqaaiabdIgaOjabdAha2bqabaGccqWGybawdaWgaaWcbaGaemODayNaemiBaWMaemizaqMaemiBaWgabeaaaqaaceqaaiabbkfasjabbggaHjabbsha0jabbwgaLjabbccaGiabb+gaVjabbAgaMjabbccaGiabbwha1jabbchaWjabbsha0jabbggaHjabbUgaRjabbwgaLbqaaiabb+gaVjabbAgaMjabbccaGiabbAfawjabbYeamjabbseaejabbYeamjabb2caTiabbkgaIjabb+gaVjabbwha1jabb6gaUjabbsgaKjabbccaGiabbgeabjabbIha4bqaaiabbMgaPjabb6gaUjabbsha0jabb+gaVjabbccaGiabbggaHjabbYgaSjabbkgaIjabbwha1jabb2gaTjabbMgaPjabb6gaUjabbccaGiabbkgaIjabbMha5jabbccaGiabbYeamjabbcfaqjabbYeambaakiaawIJ=aiabgUcaRmaayaaabaGaemOCai3aaSbaaSqaaiabdggaHjabdYgaSbqabaGccqWGybawdaWgaaWcbaGaemiBaWMaemizaqMaemiBaWgabeaaaqaaceqaaiabbkfasjabbggaHjabbsha0jabbwgaLjabbccaGiabb+gaVjabbAgaMjabbccaGiabbwha1jabbchaWjabbsha0jabbggaHjabbUgaRjabbwgaLbqaaiabb+gaVjabbAgaMjabbccaGiabbYeamjabbseaejabbYeamjabb2caTiabbkgaIjabb+gaVjabbwha1jabb6gaUjabbsgaKjabbccaGiabbgeabjabbIha4bqaaiabbkgaIjabbMha5jabbccaGiabbggaHjabbYgaSjabbkgaIjabbwha1jabb2gaTjabbMgaPjabb6gaUjabbccaGiabbggaHjabbsha0jabbccaGiabb2gaTjabbwha1jabbohaZjabbogaJjabbYgaSjabbwgaLbaakiaawIJ=aiabgkHiTmaayaaabaGaemOCai3aaSbaaSqaaiabdYgaSjabdggaHbqabaGccqWGybawdaWgaaWcbaGaemyyaegabeaaaqaaceqaaiabbkfasjabbggaHjabbsha0jabbwgaLjabbccaGiabb+gaVjabbAgaMjabbccaGiabbsgaKjabbwgaLjabbchaWjabb+gaVjabbohaZjabbMgaPjabbsha0jabbMgaPjabb+gaVjabb6gaUbqaaiabb+gaVjabbAgaMjabbccaGiabbgeabjabbIha4jabbccaGiabbMgaPjabb6gaUjabbsha0jabb+gaVjabbccaGiabbYgaSjabbMgaPjabbAha2jabbwgaLjabbkhaYbaakiaawIJ=aiabgkHiTmaayaaabaGaemOuai1aaSbaaSqaaiabd2gaTjabdggaHbqabaGccqWGtbWudaWgaaWcbaGaemyBa0MaemyyaegabeaakmaabmaabaGaemiwaG1aaSbaaSqaaiabdggaHbqabaaakiaawIcacaGLPaaaaSabaiqabaGaeeOuaiLaeeyyaeMaeeiDaqNaeeyzauMaeeiiaaIaee4Ba8MaeeOzayMaeeiiaaIaeeizaqMaeeyzauMaeeiCaaNaee4Ba8Maee4CamNaeeyAaKMaeeiDaqNaeeyAaKMaee4Ba8MaeeOBa4gabaGaee4Ba8MaeeOzayMaeeiiaaIaeeyqaeKaeeiEaGNaeeiiaaIaeeyAaKMaeeOBa4MaeeiDaqNaee4Ba8MaeeiiaaIaeeyBa0MaeeyDauNaee4CamNaee4yamMaeeiBaWMaeeyzaugaaOGaayjo+dGaeiOla4caaa@603F@

Here, *r*_*hv *_and *r*_*al *_are the relative rates of uptake from VLDL and LDL, respectively, and *r*_*la *_is the relative rate of deposition in the liver. The parameter *R*_*ma *_(*mg*·(*ml blood*)^-1^·*tu*^-1^) is the maximum relative rate of astaxanthin deposition in the muscle, which is a saturable process and is represented by the sigmoid function *S*_*ma *_[32]. The parameter *η *is explained below.

The relative rate of change of the amount of astaxanthin in the liver (*X*_*l*_(*t*)) is dependent on the uptake from blood (from chylomicron remnants, LDL, HDL and albumin), the rate of metabolism in the liver, and the rate of production of VLDL-bound astaxanthin transported back into the blood stream;

X˙l︸(mg⋅(kg liver)−1⋅tu−1)=[rlcXcmr+rllXldl+rlaXa+rlhXhdl]Bv(t)Lw(t)−1−rldXl︸Rate ofdegradationof Ax in liver−rvXl︸Rate of transportof Ax bound toVLDL into blood,
 MathType@MTEF@5@5@+=feaafiart1ev1aaatCvAUfKttLearuWrP9MDH5MBPbIqV92AaeXatLxBI9gBaebbnrfifHhDYfgasaacH8akY=wiFfYdH8Gipec8Eeeu0xXdbba9frFj0=OqFfea0dXdd9vqai=hGuQ8kuc9pgc9s8qqaq=dirpe0xb9q8qiLsFr0=vr0=vr0dc8meaabaqaciaacaGaaeqabaqabeGadaaakeaadaagaaqaaiqbdIfayzaacaWaaSbaaSqaaiabdYgaSbqabaaabaWaaeWaaeaacqWGTbqBcqWGNbWzcqGHflY1daqadaqaaiabdUgaRjabdEgaNjabbccaGiabdYgaSjabdMgaPjabdAha2jabdwgaLjabdkhaYbGaayjkaiaawMcaamaaCaaameqabaGaeyOeI0IaeGymaedaaSGaeyyXICTaemiDaqNaemyDau3aaWbaaWqabeaacqGHsislcqaIXaqmaaaaliaawIcacaGLPaaaaOGaayjo+dGaeyypa0ZaamWaaeaacqWGYbGCdaWgaaWcbaGaemiBaWMaem4yamgabeaakiabdIfaynaaBaaaleaacqWGJbWycqWGTbqBcqWGYbGCaeqaaOGaey4kaSIaemOCai3aaSbaaSqaaiabdYgaSjabdYgaSbqabaGccqWGybawdaWgaaWcbaGaemiBaWMaemizaqMaemiBaWgabeaakiabgUcaRiabdkhaYnaaBaaaleaacqWGSbaBcqWGHbqyaeqaaOGaemiwaG1aaSbaaSqaaiabdggaHbqabaGccqGHRaWkcqWGYbGCdaWgaaWcbaGaemiBaWMaemiAaGgabeaakiabdIfaynaaBaaaleaacqWGObaAcqWGKbazcqWGSbaBaeqaaaGccaGLBbGaayzxaaGaemOqai0aaSbaaSqaaiabdAha2bqabaGcdaqadaqaaiabdsha0bGaayjkaiaawMcaaiabdYeamnaaBaaaleaacqWG3bWDaeqaaOWaaeWaaeaacqWG0baDaiaawIcacaGLPaaadaahaaWcbeqaaiabgkHiTiabigdaXaaakiabgkHiTmaayaaabaGaemOCai3aaSbaaSqaaiabdYgaSjabdsgaKbqabaGccqWGybawdaWgaaWcbaGaemiBaWgabeaaaqaaceqaaiabbkfasjabbggaHjabbsha0jabbwgaLjabbccaGiabb+gaVjabbAgaMbqaaiabbsgaKjabbwgaLjabbEgaNjabbkhaYjabbggaHjabbsgaKjabbggaHjabbsha0jabbMgaPjabb+gaVjabb6gaUbqaaiabb+gaVjabbAgaMjabbccaGiabbgeabjabbIha4jabbccaGiabbMgaPjabb6gaUjabbccaGiabbYgaSjabbMgaPjabbAha2jabbwgaLjabbkhaYbaakiaawIJ=aiabgkHiTmaayaaabaGaemOCai3aaSbaaSqaaiabdAha2bqabaGccqWGybawdaWgaaWcbaGaemiBaWgabeaaaqaaceqaaiabbkfasjabbggaHjabbsha0jabbwgaLjabbccaGiabb+gaVjabbAgaMjabbccaGiabbsha0jabbkhaYjabbggaHjabb6gaUjabbohaZjabbchaWjabb+gaVjabbkhaYjabbsha0bqaaiabb+gaVjabbAgaMjabbccaGiabbgeabjabbIha4jabbccaGiabbkgaIjabb+gaVjabbwha1jabb6gaUjabbsgaKjabbccaGiabbsha0jabb+gaVbqaaiabbAfawjabbYeamjabbseaejabbYeamjabbccaGiabbMgaPjabb6gaUjabbsha0jabb+gaVjabbccaGiabbkgaIjabbYgaSjabb+gaVjabb+gaVjabbsgaKbaakiaawIJ=aiabcYcaSaaa@FB2D@

where *r*_*lh *_is the relative rate of HDL-bound astaxanthin uptake by the liver, *r*_*ld *_is the relative rate of astaxanthin degradation in the liver, and *r*_*v *_is the relative rate of transport of astaxanthin by VLDL into the blood.

The relative rate of change of the concentration of VLDL-bound astaxanthin in the blood stream (*X*_*vldl*_(*t*)) is dependent on the amount delivered by the liver, the rate of conversion into LDL, the uptake by albumin, and the transfer of astaxanthin to HDL;

X˙vldl︸(mg⋅(ml blood)−1⋅tu−1)=rvXlBv(t)−1Lw(t)−ηrhvXvldl−(1−η)rhvXvldl︸Rate of uptake ofVLDL-bound Axinto LDL-boundAx by LPL−rhvldlXvldl︸Rate of transfer ofVLDL-bound Axinto HDL-bound Ax,
 MathType@MTEF@5@5@+=feaafiart1ev1aaatCvAUfKttLearuWrP9MDH5MBPbIqV92AaeXatLxBI9gBaebbnrfifHhDYfgasaacH8akY=wiFfYdH8Gipec8Eeeu0xXdbba9frFj0=OqFfea0dXdd9vqai=hGuQ8kuc9pgc9s8qqaq=dirpe0xb9q8qiLsFr0=vr0=vr0dc8meaabaqaciaacaGaaeqabaqabeGadaaakeaadaagaaqaaiqbdIfayzaacaWaaSbaaSqaaiabdAha2jabdYgaSjabdsgaKjabdYgaSbqabaaabaWaaeWaaeaacqWGTbqBcqWGNbWzcqGHflY1daqadaqaaiabd2gaTjabdYgaSjabbccaGiabdkgaIjabdYgaSjabd+gaVjabd+gaVjabdsgaKbGaayjkaiaawMcaamaaCaaameqabaGaeyOeI0IaeGymaedaaSGaeyyXICTaemiDaqNaemyDau3aaWbaaWqabeaacqGHsislcqaIXaqmaaaaliaawIcacaGLPaaaaOGaayjo+dGaeyypa0JaemOCai3aaSbaaSqaaiabdAha2bqabaGccqWGybawdaWgaaWcbaGaemiBaWgabeaakiabdkeacnaaBaaaleaacqWG2bGDaeqaaOWaaeWaaeaacqWG0baDaiaawIcacaGLPaaadaahaaWcbeqaaiabgkHiTiabigdaXaaakiabdYeamnaaBaaaleaacqWG3bWDaeqaaOWaaeWaaeaacqWG0baDaiaawIcacaGLPaaacqGHsisliiGacqWF3oaAcqWGYbGCdaWgaaWcbaGaemiAaGMaemODayhabeaakiabdIfaynaaBaaaleaacqWG2bGDcqWGSbaBcqWGKbazcqWGSbaBaeqaaOGaeyOeI0YaaGbaaeaadaqadaqaaiabigdaXiabgkHiTiab=D7aObGaayjkaiaawMcaaiabdkhaYnaaBaaaleaacqWGObaAcqWG2bGDaeqaaOGaemiwaG1aaSbaaSqaaiabdAha2jabdYgaSjabdsgaKjabdYgaSbqabaaaeaGabeaacqqGsbGucqqGHbqycqqG0baDcqqGLbqzcqqGGaaicqqGVbWBcqqGMbGzcqqGGaaicqqG1bqDcqqGWbaCcqqG0baDcqqGHbqycqqGRbWAcqqGLbqzcqqGGaaicqqGVbWBcqqGMbGzaeaacqqGwbGvcqqGmbatcqqGebarcqqGmbatcqqGTaqlcqqGIbGycqqGVbWBcqqG1bqDcqqGUbGBcqqGKbazcqqGGaaicqqGbbqqcqqG4baEaeaacqqGPbqAcqqGUbGBcqqG0baDcqqGVbWBcqqGGaaicqqGmbatcqqGebarcqqGmbatcqqGTaqlcqqGIbGycqqGVbWBcqqG1bqDcqqGUbGBcqqGKbazaeaacqqGbbqqcqqG4baEcqqGGaaicqqGIbGycqqG5bqEcqqGGaaicqqGmbatcqqGqbaucqqGmbataaGccaGL44pacqGHsisldaagaaqaaiabdkhaYnaaBaaaleaacqWGObaAcqWG2bGDcqWGSbaBcqWGKbazcqWGSbaBaeqaaOGaemiwaG1aaSbaaSqaaiabdAha2jabdYgaSjabdsgaKjabdYgaSbqabaaaeaGabeaacqqGsbGucqqGHbqycqqG0baDcqqGLbqzcqqGGaaicqqGVbWBcqqGMbGzcqqGGaaicqqG0baDcqqGYbGCcqqGHbqycqqGUbGBcqqGZbWCcqqGMbGzcqqGLbqzcqqGYbGCcqqGGaaicqqGVbWBcqqGMbGzaeaacqqGwbGvcqqGmbatcqqGebarcqqGmbatcqqGTaqlcqqGIbGycqqGVbWBcqqG1bqDcqqGUbGBcqqGKbazcqqGGaaicqqGbbqqcqqG4baEaeaacqqGPbqAcqqGUbGBcqqG0baDcqqGVbWBcqqGGaaicqqGibascqqGebarcqqGmbatcqqGTaqlcqqGIbGycqqGVbWBcqqG1bqDcqqGUbGBcqqGKbazcqqGGaaicqqGbbqqcqqG4baEaaGccaGL44pacqGGSaalaaa@16C4@

where *r*_*hv *_is the relative rate of conversion of VLDL-bound astaxanthin into LDL-bound astaxanthin as well as the accompanying uptake into the albumin, due to LPL hydrolysis. The fraction of VLDL astaxanthin that is taken up by albumin is represented by *η*, (0 ≤ *η *≤ 1). The parameter *r*_*hvldl *_is the transfer of astaxanthin to HDL by the action of CETP.

The relative rate of change of astaxanthin concentration in LDL (*X*_*ldl*_(*t*)) is expressed in terms of the conversion of VLDL-bound astaxanthin into LDL, the uptake into albumin at the muscle, the transfer into HDL and the uptake into the liver;

X˙ldl︸(mg⋅(ml blood)−1⋅tu−1)=(1−η)rhvXvldl−ralXldl︸Rate of uptakeof LDL-boundAx into albuminat muscle−rhldlXldl︸Rate of transfer ofLDL-bound Ax intoHDL-bound Ax−rllXldl,
 MathType@MTEF@5@5@+=feaafiart1ev1aaatCvAUfKttLearuWrP9MDH5MBPbIqV92AaeXatLxBI9gBaebbnrfifHhDYfgasaacH8akY=wiFfYdH8Gipec8Eeeu0xXdbba9frFj0=OqFfea0dXdd9vqai=hGuQ8kuc9pgc9s8qqaq=dirpe0xb9q8qiLsFr0=vr0=vr0dc8meaabaqaciaacaGaaeqabaqabeGadaaakeaadaagaaqaaiqbdIfayzaacaWaaSbaaSqaaiabdYgaSjabdsgaKjabdYgaSbqabaaabaWaaeWaaeaacqWGTbqBcqWGNbWzcqGHflY1daqadaqaaiabd2gaTjabdYgaSjabbccaGiabdkgaIjabdYgaSjabd+gaVjabd+gaVjabdsgaKbGaayjkaiaawMcaamaaCaaameqabaGaeyOeI0IaeGymaedaaSGaeyyXICTaemiDaqNaemyDau3aaWbaaWqabeaacqGHsislcqaIXaqmaaaaliaawIcacaGLPaaaaOGaayjo+dGaeyypa0ZaaeWaaeaacqaIXaqmcqGHsisliiGacqWF3oaAaiaawIcacaGLPaaacqWGYbGCdaWgaaWcbaGaemiAaGMaemODayhabeaakiabdIfaynaaBaaaleaacqWG2bGDcqWGSbaBcqWGKbazcqWGSbaBaeqaaOGaeyOeI0YaaGbaaeaacqWGYbGCdaWgaaWcbaGaemyyaeMaemiBaWgabeaakiabdIfaynaaBaaaleaacqWGSbaBcqWGKbazcqWGSbaBaeqaaaabaiqabaGaeeOuaiLaeeyyaeMaeeiDaqNaeeyzauMaeeiiaaIaee4Ba8MaeeOzayMaeeiiaaIaeeyDauNaeeiCaaNaeeiDaqNaeeyyaeMaee4AaSMaeeyzaugabaGaee4Ba8MaeeOzayMaeeiiaaIaeeitaWKaeeiraqKaeeitaWKaeeyla0IaeeOyaiMaee4Ba8MaeeyDauNaeeOBa4MaeeizaqgabaGaeeyqaeKaeeiEaGNaeeiiaaIaeeyAaKMaeeOBa4MaeeiDaqNaee4Ba8MaeeiiaaIaeeyyaeMaeeiBaWMaeeOyaiMaeeyDauNaeeyBa0MaeeyAaKMaeeOBa4gabaGaeeyyaeMaeeiDaqNaeeiiaaIaeeyBa0MaeeyDauNaee4CamNaee4yamMaeeiBaWMaeeyzaugaaOGaayjo+dGaeyOeI0YaaGbaaeaacqWGYbGCdaWgaaWcbaGaemiAaGMaemiBaWMaemizaqMaemiBaWgabeaakiabdIfaynaaBaaaleaacqWGSbaBcqWGKbazcqWGSbaBaeqaaaabaiqabaGaeeOuaiLaeeyyaeMaeeiDaqNaeeyzauMaeeiiaaIaee4Ba8MaeeOzayMaeeiiaaIaeeiDaqNaeeOCaiNaeeyyaeMaeeOBa4Maee4CamNaeeOzayMaeeyzauMaeeOCaiNaeeiiaaIaee4Ba8MaeeOzaygabaGaeeitaWKaeeiraqKaeeitaWKaeeyla0IaeeOyaiMaee4Ba8MaeeyDauNaeeOBa4MaeeizaqMaeeiiaaIaeeyqaeKaeeiEaGNaeeiiaaIaeeyAaKMaeeOBa4MaeeiDaqNaee4Ba8gabaGaeeisaGKaeeiraqKaeeitaWKaeeyla0IaeeOyaiMaee4Ba8MaeeyDauNaeeOBa4MaeeizaqMaeeiiaaIaeeyqaeKaeeiEaGhaaOGaayjo+dGaeyOeI0IaemOCai3aaSbaaSqaaiabdYgaSjabdYgaSbqabaGccqWGybawdaWgaaWcbaGaemiBaWMaemizaqMaemiBaWgabeaakiabcYcaSaaa@0336@

where *r*_*al *_is the relative rate of astaxanthin uptake in the muscle and *r*_*hldl *_is the relative transfer rate into HDL due to CETP activity.

The relative rate of change of astaxanthin concentration in HDL (*X*_*hdl*_(*t*)) is represented as the transfer of astaxanthin from VLDL and LDL, the uptake into the muscle during maturation and the delivery of astaxanthin to the liver;

X˙hdl︸(mg⋅(ml blood)−1⋅tu−1)=rhldlXldl+rhvldlXvldl+rhmHsexmatXmBv(t)−1Mw(t)︸Rate of transferof Ax into HDLfrom the muscle−rlhXhdl,
 MathType@MTEF@5@5@+=feaafiart1ev1aaatCvAUfKttLearuWrP9MDH5MBPbIqV92AaeXatLxBI9gBaebbnrfifHhDYfgasaacH8akY=wiFfYdH8Gipec8Eeeu0xXdbba9frFj0=OqFfea0dXdd9vqai=hGuQ8kuc9pgc9s8qqaq=dirpe0xb9q8qiLsFr0=vr0=vr0dc8meaabaqaciaacaGaaeqabaqabeGadaaakeaadaagaaqaaiqbdIfayzaacaWaaSbaaSqaaiabdIgaOjabdsgaKjabdYgaSbqabaaabaWaaeWaaeaacqWGTbqBcqWGNbWzcqGHflY1daqadaqaaiabd2gaTjabdYgaSjabbccaGiabdkgaIjabdYgaSjabd+gaVjabd+gaVjabdsgaKbGaayjkaiaawMcaamaaCaaameqabaGaeyOeI0IaeGymaedaaSGaeyyXICTaemiDaqNaemyDau3aaWbaaWqabeaacqGHsislcqaIXaqmaaaaliaawIcacaGLPaaaaOGaayjo+dGaeyypa0JaemOCai3aaSbaaSqaaiabdIgaOjabdYgaSjabdsgaKjabdYgaSbqabaGccqWGybawdaWgaaWcbaGaemiBaWMaemizaqMaemiBaWgabeaakiabgUcaRiabdkhaYnaaBaaaleaacqWGObaAcqWG2bGDcqWGSbaBcqWGKbazcqWGSbaBaeqaaOGaemiwaG1aaSbaaSqaaiabdAha2jabdYgaSjabdsgaKjabdYgaSbqabaGccqGHRaWkdaagaaqaaiabdkhaYnaaBaaaleaacqWGObaAcqWGTbqBaeqaaOGaemisaG0aaSbaaSqaaiabdohaZjabdwgaLjabdIha4jabd2gaTjabdggaHjabdsha0bqabaGccqWGybawdaWgaaWcbaGaemyBa0gabeaakiabdkeacnaaBaaaleaacqWG2bGDaeqaaOWaaeWaaeaacqWG0baDaiaawIcacaGLPaaadaahaaWcbeqaaiabgkHiTiabigdaXaaakiabd2eannaaBaaaleaacqWG3bWDaeqaaOWaaeWaaeaacqWG0baDaiaawIcacaGLPaaaaSabaiqabaGaeeOuaiLaeeyyaeMaeeiDaqNaeeyzauMaeeiiaaIaee4Ba8MaeeOzayMaeeiiaaIaeeiDaqNaeeOCaiNaeeyyaeMaeeOBa4Maee4CamNaeeOzayMaeeyzauMaeeOCaihabaGaee4Ba8MaeeOzayMaeeiiaaIaeeyqaeKaeeiEaGNaeeiiaaIaeeyAaKMaeeOBa4MaeeiDaqNaee4Ba8MaeeiiaaIaeeisaGKaeeiraqKaeeitaWeabaGaeeOzayMaeeOCaiNaee4Ba8MaeeyBa0MaeeiiaaIaeeiDaqNaeeiAaGMaeeyzauMaeeiiaaIaeeyBa0MaeeyDauNaee4CamNaee4yamMaeeiBaWMaeeyzaugaaOGaayjo+dGaeyOeI0IaemOCai3aaSbaaSqaaiabdYgaSjabdIgaObqabaGccqWGybawdaWgaaWcbaGaemiAaGMaemizaqMaemiBaWgabeaakiabcYcaSaaa@D312@

where *r*_*hm *_is the relative rate of uptake of astaxanthin from the muscle and *H*_*sexmat *_denotes the status of sexual maturation of the salmon. It has a value of 0 in an immature fish and 1 in a sexually mature one.

The relative rate of change of concentration of astaxanthin bound to muscle (*X*_*m*_(*t*)) is equal to the rate of astaxanthin taken up from the blood via the albumin minus the rate of active transport out of the muscle by the HDL, to gonads and skin;

X˙m︸(mg⋅(kg muscle)−1⋅tu−1)=RmaSma(Xa)Bv(t)Mw(t)−1−rhmHsexmatXm,
 MathType@MTEF@5@5@+=feaafiart1ev1aaatCvAUfKttLearuWrP9MDH5MBPbIqV92AaeXatLxBI9gBaebbnrfifHhDYfgasaacH8akY=wiFfYdH8Gipec8Eeeu0xXdbba9frFj0=OqFfea0dXdd9vqai=hGuQ8kuc9pgc9s8qqaq=dirpe0xb9q8qiLsFr0=vr0=vr0dc8meaabaqaciaacaGaaeqabaqabeGadaaakeaadaagaaqaaiqbdIfayzaacaWaaSbaaSqaaiabd2gaTbqabaaabaWaaeWaaeaacqWGTbqBcqWGNbWzcqGHflY1daqadaqaaiabdUgaRjabdEgaNjabbccaGiabd2gaTjabdwha1jabdohaZjabdogaJjabdYgaSjabdwgaLbGaayjkaiaawMcaamaaCaaameqabaGaeyOeI0IaeGymaedaaSGaeyyXICTaemiDaqNaemyDau3aaWbaaWqabeaacqGHsislcqaIXaqmaaaaliaawIcacaGLPaaaaOGaayjo+dGaeyypa0JaemOuai1aaSbaaSqaaiabd2gaTjabdggaHbqabaGccqWGtbWudaWgaaWcbaGaemyBa0MaemyyaegabeaakmaabmaabaGaemiwaG1aaSbaaSqaaiabdggaHbqabaaakiaawIcacaGLPaaacqWGcbGqdaWgaaWcbaGaemODayhabeaakmaabmaabaGaemiDaqhacaGLOaGaayzkaaGaemyta00aaSbaaSqaaiabdEha3bqabaGcdaqadaqaaiabdsha0bGaayjkaiaawMcaamaaCaaaleqabaGaeyOeI0IaeGymaedaaOGaeyOeI0IaemOCai3aaSbaaSqaaiabdIgaOjabd2gaTbqabaGccqWGibasdaWgaaWcbaGaem4CamNaemyzauMaemiEaGNaemyBa0MaemyyaeMaemiDaqhabeaakiabdIfaynaaBaaaleaacqWGTbqBaeqaaOGaeiilaWcaaa@7C4B@

where the relative transport rate of astaxanthin out of the muscle *r*_*hm *_is dependent on the rate of degradation in the muscle.

#### b. Sexual maturation and spawning migration

In order to test the hypothesis behind the life history reasoning, we add two new variables to represent astaxanthin concentration in the skin (*X*_*s*_(*t*)) and gonads (*X*_*g*_(*t*) during spawning migration. The variable *H*_*sexmat *_is now set to 1, enabling the flow of astaxanthin from the muscle into the HDL, and from there into the skin and gonads. The fraction of pigment redistributed to the gonads is represented as *ω*, the rest of which (1-*ω*) goes to the skin. The HDL equation is changed into:

X˙hdl=rhldlXldl+rhvldlXvldl+rhmHsexmatXmBv(t)−1Mw(t)︸Rate of uptake of Axfrom muscle−rlhXhdl−ωrgsXhdlHsexmat︸Rate of uptake into gonads−(1−ω)rgsXhdlHsexmat︸Rate of uptake into skin.
 MathType@MTEF@5@5@+=feaafiart1ev1aaatCvAUfKttLearuWrP9MDH5MBPbIqV92AaeXatLxBI9gBaebbnrfifHhDYfgasaacH8akY=wiFfYdH8Gipec8Eeeu0xXdbba9frFj0=OqFfea0dXdd9vqai=hGuQ8kuc9pgc9s8qqaq=dirpe0xb9q8qiLsFr0=vr0=vr0dc8meaabaqaciaacaGaaeqabaqabeGadaaakeaacuWGybawgaGaamaaBaaaleaacqWGObaAcqWGKbazcqWGSbaBaeqaaOGaeyypa0JaemOCai3aaSbaaSqaaiabdIgaOjabdYgaSjabdsgaKjabdYgaSbqabaGccqWGybawdaWgaaWcbaGaemiBaWMaemizaqMaemiBaWgabeaakiabgUcaRiabdkhaYnaaBaaaleaacqWGObaAcqWG2bGDcqWGSbaBcqWGKbazcqWGSbaBaeqaaOGaemiwaG1aaSbaaSqaaiabdAha2jabdYgaSjabdsgaKjabdYgaSbqabaGccqGHRaWkdaagaaqaaiabdkhaYnaaBaaaleaacqWGObaAcqWGTbqBaeqaaOGaemisaG0aaSbaaSqaaiabdohaZjabdwgaLjabdIha4jabd2gaTjabdggaHjabdsha0bqabaGccqWGybawdaWgaaWcbaGaemyBa0gabeaakiabdkeacnaaBaaaleaacqWG2bGDaeqaaOWaaeWaaeaacqWG0baDaiaawIcacaGLPaaadaahaaWcbeqaaiabgkHiTiabigdaXaaakiabd2eannaaBaaaleaacqWG3bWDaeqaaOWaaeWaaeaacqWG0baDaiaawIcacaGLPaaaaSabaiqabaGaeeOuaiLaeeyyaeMaeeiDaqNaeeyzauMaeeiiaaIaee4Ba8MaeeOzayMaeeiiaaIaeeyDauNaeeiCaaNaeeiDaqNaeeyyaeMaee4AaSMaeeyzauMaeeiiaaIaee4Ba8MaeeOzayMaeeiiaaIaeeyqaeKaeeiEaGhabaGaeeOzayMaeeOCaiNaee4Ba8MaeeyBa0MaeeiiaaIaeeyBa0MaeeyDauNaee4CamNaee4yamMaeeiBaWMaeeyzaugaaOGaayjo+dGaeyOeI0IaemOCai3aaSbaaSqaaiabdYgaSjabdIgaObqabaGccqWGybawdaWgaaWcbaGaemiAaGMaemizaqMaemiBaWgabeaakiabgkHiTmaayaaabaacciGae8xYdCNaemOCai3aaSbaaSqaaiabdEgaNjabdohaZbqabaGccqWGybawdaWgaaWcbaGaemiAaGMaemizaqMaemiBaWgabeaakiabdIeainaaBaaaleaacqWGZbWCcqWGLbqzcqWG4baEcqWGTbqBcqWGHbqycqWG0baDaeqaaaqaaiabbkfasjabbggaHjabbsha0jabbwgaLjabbccaGiabb+gaVjabbAgaMjabbccaGiabbwha1jabbchaWjabbsha0jabbggaHjabbUgaRjabbwgaLjabbccaGiabbMgaPjabb6gaUjabbsha0jabb+gaVjabbccaGiabbEgaNjabb+gaVjabb6gaUjabbggaHjabbsgaKjabbohaZbGccaGL44pacqGHsisldaagaaqaamaabmaabaGaeGymaeJaeyOeI0Iae8xYdChacaGLOaGaayzkaaGaemOCai3aaSbaaSqaaiabdEgaNjabdohaZbqabaGccqWGybawdaWgaaWcbaGaemiAaGMaemizaqMaemiBaWgabeaakiabdIeainaaBaaaleaacqWGZbWCcqWGLbqzcqWG4baEcqWGTbqBcqWGHbqycqWG0baDaeqaaaqaaiabbkfasjabbggaHjabbsha0jabbwgaLjabbccaGiabb+gaVjabbAgaMjabbccaGiabbwha1jabbchaWjabbsha0jabbggaHjabbUgaRjabbwgaLjabbccaGiabbMgaPjabb6gaUjabbsha0jabb+gaVjabbccaGiabbohaZjabbUgaRjabbMgaPjabb6gaUbGccaGL44pacqGGUaGlaaa@1742@

Two new equations are added to represent skin and gonad uptake;

X˙s︸(mg⋅(kg skin)−1⋅tu−1)=(1−ω)rgsXhdlHsexmatBv(t)Sw(t)−1,X˙g︸(mg⋅(kg gonad)−1⋅tu−1)=ωrgsXhdlHsexmatBv(t)Gw(t)−1.
 MathType@MTEF@5@5@+=feaafiart1ev1aaatCvAUfKttLearuWrP9MDH5MBPbIqV92AaeXatLxBI9gBaebbnrfifHhDYfgasaacH8akY=wiFfYdH8Gipec8Eeeu0xXdbba9frFj0=OqFfea0dXdd9vqai=hGuQ8kuc9pgc9s8qqaq=dirpe0xb9q8qiLsFr0=vr0=vr0dc8meaabaqaciaacaGaaeqabaqabeGadaaakqaabeqaamaayaaabaGafmiwaGLbaiaadaWgaaWcbaGaem4Camhabeaaaeaadaqadaqaaiabd2gaTjabdEgaNjabgwSixpaabmaabaGaem4AaSMaem4zaCMaeeiiaaIaem4CamNaem4AaSMaemyAaKMaemOBa4gacaGLOaGaayzkaaWaaWbaaWqabeaacqGHsislcqaIXaqmaaWccqGHflY1cqWG0baDcqWG1bqDdaahaaadbeqaaiabgkHiTiabigdaXaaaaSGaayjkaiaawMcaaaGccaGL44pacqGH9aqpdaqadaqaaiabigdaXiabgkHiTGGaciab=L8a3bGaayjkaiaawMcaaiabdkhaYnaaBaaaleaacqWGNbWzcqWGZbWCaeqaaOGaemiwaG1aaSbaaSqaaiabdIgaOjabdsgaKjabdYgaSbqabaGccqWGibasdaWgaaWcbaGaem4CamNaemyzauMaemiEaGNaemyBa0MaemyyaeMaemiDaqhabeaakiabdkeacnaaBaaaleaacqWG2bGDaeqaaOWaaeWaaeaacqWG0baDaiaawIcacaGLPaaacqWGtbWudaWgaaWcbaGaem4DaChabeaakmaabmaabaGaemiDaqhacaGLOaGaayzkaaWaaWbaaSqabeaacqGHsislcqaIXaqmaaGccqGGSaalaeaadaagaaqaaiqbdIfayzaacaWaaSbaaSqaaiabdEgaNbqabaaabaWaaeWaaeaacqWGTbqBcqWGNbWzcqGHflY1daqadaqaaiabdUgaRjabdEgaNjabbccaGiabdEgaNjabd+gaVjabd6gaUjabdggaHjabdsgaKbGaayjkaiaawMcaamaaCaaameqabaGaeyOeI0IaeGymaedaaSGaeyyXICTaemiDaqNaemyDau3aaWbaaWqabeaacqGHsislcqaIXaqmaaaaliaawIcacaGLPaaaaOGaayjo+dGaeyypa0Jae8xYdCNaemOCai3aaSbaaSqaaiabdEgaNjabdohaZbqabaGccqWGybawdaWgaaWcbaGaemiAaGMaemizaqMaemiBaWgabeaakiabdIeainaaBaaaleaacqWGZbWCcqWGLbqzcqWG4baEcqWGTbqBcqWGHbqycqWG0baDaeqaaOGaemOqai0aaSbaaSqaaiabdAha2bqabaGcdaqadaqaaiabdsha0bGaayjkaiaawMcaaiabdEeahnaaBaaaleaacqWG3bWDaeqaaOWaaeWaaeaacqWG0baDaiaawIcacaGLPaaadaahaaWcbeqaaiabgkHiTiabigdaXaaakiabc6caUaaaaa@B970@

### Calibration of the model/constraints to be accounted for

Though the values for most of the parameters of the model are still unknown, the significant amount of research done in the area of pigment deposition in salmonids provided a number of constraints for the model. We know that the optimal astaxanthin dietary content for Atlantic salmon is around 60 mg/kg feed [8] out of which only around 15% is retained in the flesh [56]. The mean carotenoid content in salmon muscle is around 7 mg/kg, though higher values have been observed as well. The uptake from the intestine into the blood takes between 18 to 30 hrs [18,19], with the maximum carotenoid concentration in the blood occurring around 30 hrs. In the blood, about 50% is attached to serum albumin compared to the lipoproteins [12].

There is a metabolic reduction and degradation in the liver [57]. The uptake into the liver is saturable but only at very high doses [29].

The uptake in the muscle cells is saturable [43] and the astaxanthin level is not affected by starvation up to 85 days in the immature fish [36,37], indicating a negligible degradation.

In the sexually mature fish, the total whole-body carotenoid content of females and males were 73–79% and 18–19% of that in the immature fish, respectively [38]. In males the skin content increased strongly while in females the pigment levels in the gonads and eggs increased.

Using these constraints, as well as those provided by the experimental data, we assigned values to the different parameters (Table 1).

Given the fact that our experimental data has two different time scales (hours and days) as well as various other differences in the experimental set-up (temperature, fish weight, feed etc.) we do not perform any specific computational fitting procedures on it. At this stage, such an exercise would not reveal anything of significance as regards to the model setup. Later, the model will be refined to deal with specific sub-systems (e.g. muscle uptake) and in that case we will carry out more rigorous parameter estimations.

### Sensitivity analysis – QSS

Setting the derivatives of the model equations equal to zero, we arrive at a number of easy-to-solve linear equations and one equation that is not generally solvable, the equation for the QSS level of astaxanthin in albumin;

*Ax*_*a *_+ *S*_*ma*_(*x*_*a*_;1) = *BI*_*axi*_,    (1)

where *x*_*a *_= *X*_*a*_/*θ*_*ma *_is the albumin astaxanthin level relative to the threshold of muscle uptake and

A=αrlaRma,B=(1−α(1−ϕ))rcRmaBvθma,α=rldrld+β rv,β=rhvrhv+rhvldlral+η (rll+rhldl)ral+rll+rhldl.     (2)
 MathType@MTEF@5@5@+=feaafiart1ev1aaatCvAUfKttLearuWrP9MDH5MBPbIqV92AaeXatLxBI9gBaebbnrfifHhDYfgasaacH8akY=wiFfYdH8Gipec8Eeeu0xXdbba9frFj0=OqFfea0dXdd9vqai=hGuQ8kuc9pgc9s8qqaq=dirpe0xb9q8qiLsFr0=vr0=vr0dc8meaabaqaciaacaGaaeqabaqabeGadaaakeaafaqadeabbaaaaeaacqWGbbqqcqGH9aqpiiGacqWFXoqydaWcaaqaaiabdkhaYnaaBaaaleaacqWGSbaBcqWGHbqyaeqaaaGcbaGaemOuai1aaSbaaSqaaiabd2gaTjabdggaHbqabaaaaOGaeiilaWcabaGaemOqaiKaeyypa0ZaaeWaaeaacqaIXaqmcqGHsislcqWFXoqydaqadaqaaiabigdaXiabgkHiTiab=v9aQbGaayjkaiaawMcaaaGaayjkaiaawMcaamaalaaabaGaemOCai3aaSbaaSqaaiabdogaJbqabaaakeaacqWGsbGudaWgaaWcbaGaemyBa0MaemyyaegabeaakiabdkeacnaaBaaaleaacqWG2bGDaeqaaOGae8hUde3aaSbaaSqaaiabd2gaTjabdggaHbqabaaaaOGaeiilaWcabaGae8xSdeMaeyypa0ZaaSaaaeaacqWGYbGCdaWgaaWcbaGaemiBaWMaemizaqgabeaaaOqaaiabdkhaYnaaBaaaleaacqWGSbaBcqWGKbazaeqaaOGaey4kaSIaeqOSdiMaeeiiaaIaemOCai3aaSbaaSqaaiabdAha2bqabaaaaOGaeiilaWcabaGaeqOSdiMaeyypa0ZaaSaaaeaacqWGYbGCdaWgaaWcbaGaemiAaGMaemODayhabeaaaOqaaiabdkhaYnaaBaaaleaacqWGObaAcqWG2bGDaeqaaOGaey4kaSIaemOCai3aaSbaaSqaaiabdIgaOjabdAha2jabdYgaSjabdsgaKjabdYgaSbqabaaaaOWaaSaaaeaacqWGYbGCdaWgaaWcbaGaemyyaeMaemiBaWgabeaakiabgUcaRiabeE7aOjabbccaGmaabmaabaGaemOCai3aaSbaaSqaaiabdYgaSjabdYgaSbqabaGccqGHRaWkcqWGYbGCdaWgaaWcbaGaemiAaGMaemiBaWMaemizaqMaemiBaWgabeaaaOGaayjkaiaawMcaaaqaaiabdkhaYnaaBaaaleaacqWGHbqycqWGSbaBaeqaaOGaey4kaSIaemOCai3aaSbaaSqaaiabdYgaSjabdYgaSbqabaGccqGHRaWkcqWGYbGCdaWgaaWcbaGaemiAaGMaemiBaWMaemizaqMaemiBaWgabeaaaaGccqGGUaGlaaGaaCzcaiaaxMaadaqadaqaaiabikdaYaGaayjkaiaawMcaaaaa@A89E@

The relationship between the meta-parameters *A *and *B *and the solution *x*_*a *_of Eq. (1) is examined analytically. By implicit differentiation of this equation we find that *x*_*a *_increases with *B *and decreases with *A *(when *A *and *B *are fixed, respectively). As *S*_*ma *_is a monotonically increasing function of *x*_*a*_, *y *= *S*_*ma*_(*x*_*a*_;1) is also increasing with *B *and decreasing with *A*. Below we investigate the sensitivity of the muscle uptake rate (MUR) *R*_*ma*_*S*_*ma*_(*x*_*a*_;1) to variation in the parameters that constitute *A *and *B*. For each chosen parameter *P*, all other parameters are fixed to their values from the multiple-feeding experiment, and we consider the values *P *= 2^*k *^*P*_0_, *k *= -*n*,.., -1, 0, 1,.., *n *where *P*_0 _is the value of *P *given in Table 1. As a result, a profile representing the sensitivity of the MUR to variation in the chosen parameter is obtained.

#### a. Liver uptake rate *r*_*la *_and intestinal uptake rate *r*_*c*_

We note from Eq. (2) that *A* and *r*_*la *_as well as *B *and *r*_*c *_are proportional. This suggests that the MUR is increasing with *r*_*c *_and decreasing with *r*_*la *_(Figures 4A and 4B).

#### b. Liver metabolism rate *r*_*ld *_and uptake rate *r*_*ll*_

The meta-meta-parameter *α *is positively related to *A *and negatively related to *B *enabling us to conclude that the solution *x*_*a *_as well as MUR decrease with *α*. Due to the hyperbolic relationship between the liver metabolism rate *r*_*ld *_and *α*, the MUR thus decreases with *r*_*ld*_. Moreover *α *is a decreasing function of another meta-meta-parameter, *β*, which is a slightly decreasing function of the liver uptake rate *r*_*ll*_. Thus, since *α *increases with *r*_*ll*_, the MUR decreases with *r*_*ll*. _The negative relationship between the MUR and *r*_*ll *_and *r*_*ld *_is depicted in Figure 4C and 4D, respectively.

From Figures 4C and 4D we observe that the decrease of the MUR with increasing *r*_*ll *_and *r*_*ld *_is negligible (note the scaling of the vertical axis). This is due to the following: Variation in *r*_*ll *_causes minute changes in the MUR since whether *r*_*ll *_is zero or infinite, *β *remains within a narrow subinterval (*how *narrow is of course parameter value dependent) of [0, 1]. In turn, *α*, and to an even larger extent, the MUR, are virtually unaffected by changes in *r*_*ll*_. Similarly, since α is also confined to the unit interval, *A *and *B *cannot attain values outside the intervals [0, *r*_*la*_/*R*_*ma*_] and [*φr*_*c*_/(*R*_*ma*_*B*_*v*_*θ*_*ma*_), *r*_*c*_/(*R*_*ma*_*B*_*v*_*θ*_*ma*_)], respectively. Thus for a wide range of parameter values we expect that the sensitivity of the MUR to variation in *r*_*ld *_is also small. We note that the liver uptake rates *r*_*lc *_and *r*_*lh *_do not affect the QSS of *x*_*a *_at all; hence the MUR is independent of these rates.

#### c. Muscle uptake rate *R*_*ma *_and threshold *θ*_*ma*_

Both meta-parameters *A *and *B *are inversely related to the maximum muscle uptake rate *R*_*ma*_. Using implicit differentiation we conclude that *x*_*a *_is decreasing with *R*_*ma*_. In contrast, the derivative of *y *= *R*_*ma*_*S*_*ma*_(*x*_*a*_;1) with respect to *R*_*ma *_is positive;

dydRma=yRmaAA+dS/dxa,
 MathType@MTEF@5@5@+=feaafiart1ev1aaatCvAUfKttLearuWrP9MDH5MBPbIqV92AaeXatLxBI9gBaebbnrfifHhDYfgasaacH8akY=wiFfYdH8Gipec8Eeeu0xXdbba9frFj0=OqFfea0dXdd9vqai=hGuQ8kuc9pgc9s8qqaq=dirpe0xb9q8qiLsFr0=vr0=vr0dc8meaabaqaciaacaGaaeqabaqabeGadaaakeaadaWcaaqaaiabdsgaKnaaBaaaleaacqWG5bqEaeqaaaGcbaGaemizaqMaemOuai1aaSbaaSqaaiabd2gaTjabdggaHbqabaaaaOGaeyypa0ZaaSaaaeaacqWG5bqEaeaacqWGsbGudaWgaaWcbaGaemyBa0MaemyyaegabeaaaaGcdaWcaaqaaiabdgeabbqaaiabdgeabjabgUcaRiabdsgaKjabdofatjabc+caViabdsgaKjabdIha4naaBaaaleaacqWGHbqyaeqaaaaakiabcYcaSaaa@475B@

i.e. the MUR is an increasing function of *R*_*ma *_(Figure 4E). Finally, the muscle uptake threshold *θ*_*ma *_and *B *are negatively related, hence the MUR decreases with *θ*_*ma *_(Figure 4F).

### Sensitivity analysis – time-series

The sensitivity analysis on the single pulse feeding data was performed by varying the 17 individual parameter values (all the rates and the *μ*_2 _and *σ*_2 _terms of the integral kernel *I*_*axi*_) over a uniformly distributed interval centred on the values given in Table 1. Following simulation of the model with the random values, the overall relative sum of squared deviations from the experimental data was calculated as well as the relative deviation at each experimental time point. The simulations were performed 50,000 times and we chose those parameter sets which gave an output having a total deviation of 30% or less from the experimental data, as well as a relative deviation of 50% or less at each individual experimental time point. The range of values for each individual parameter in the selected parameter sets (1,264) were then plotted separately (Figure 5A). As can be seen from the figure, the parameters *r*_*c *_and the mean *μ*_2 _and standard deviation *σ*_2 _of the integral kernel have the smallest range. The distribution of these three values is not uniform (Figure 5B) even though the original variation was drawn from a uniform distribution. However, even for this selected set of parameter values giving the best fit, the distribution of the rest of the 14 parameters is approximately uniform and thus of no interest to us. The ratio of these three parameters to the rest was calculated for all the selected sets, as well as for the total 50,000 simulations. There is a significant narrowing in the range of values giving a good fit, for the interaction between *r*_*c *_and *μ*_2 _(Figure 5C) as well as for the ratio between *μ*_2 _and *σ*_2 _(Figure 5D).

## Authors' contributions

HR carried out the model development, simulations and manuscript drafting. LØ participated in the model development and performed the sensitivity analysis. DIV helped in model development. SWO conceived of the study, and participated in its design and coordination and helped to draft the manuscript. All authors read and approved the final manuscript.

## Figures and Tables

**Table 1:** Values of the model parameters

**Relative rates**	**Values (*per day*)**
*r*_*hc *_– transfer from chylomicrons to remnants and albumin	0.5
*r*_*lc *_– uptake from chylomicron remnant to liver	5
*r*_*la *_– deposition in liver from albumin	1.2
*r*_*ll *_– uptake into liver from LDL	5
*r*_*lh *_– uptake into liver from HDL	5
*r*_*ld *_– enzymatic degradation and excretion in liver	0.5
*r*_*v *_– uptake by VLDL from liver	0.25
*r*_*hv *_– transfer from VLDL to LDL and albumin	0.5
*r*_*hvldl *_– uptake into HDL from VLDL	0.5
*r*_*al *_– uptake into albumin from LDL at muscle	0.05
*r*_*hldl *_– uptake into HDL from LDL	0.05
*R*_*ma *_– deposition in muscle from albumin	0.00024 *mg*·(*ml blood*)^-1^·*day*^-1^
*r*_*hm *_– transfer into HDL from muscle	0.03
*r*_*c *_– bioavailable fraction of astaxanthin fed	0.5

**Tissue weight constants**	**Values**

*b *– blood volume as a fraction of fish weight	0.045 *litre blood*·(*kg fish*)^-1^
*l *– liver weight as a fraction of fish weight	0.01 *kg liver*·(*kg fish*)^-1^
*m *– muscle weight as a fraction of fish weight	0.50 *kg muscle*·(*kg fish*)^-1^
*γ*, *λ*, *ρ *– allometric constants	1

**Sigmoid values**	**Values**

*n*_*ma *_– Hill coefficient	2
*θ*_*ma *_– threshold	0.00005 *mg*·(*ml blood*)^-1^

**Fractions taken up by albumin**	**Values**

*φ *– from chylomicrons	0.75
*η *– from VLDL	0.75

**Convolution terms**	**Values**

*μ*_1 _– Mean of *I*_*ax*_	1
*σ*_1 _– Standard deviations of *I*_*ax*_	0.001
*μ*_2 _– Mean of *Z*	20
*σ*_2 _– Standard deviations of *Z*	12
